# Methaemoglobinaemia Associated With Mixed Cocaine and Amphetamine Overdose: A Case Report

**DOI:** 10.7759/cureus.51748

**Published:** 2024-01-06

**Authors:** Mohamed Eshmandi, Ahmed Mohamed, Belal Khalil, Alla Belhaj

**Affiliations:** 1 Anaesthesiology, Southend University Hospital, Mid and South Essex NHS Foundation Trust, Southend-on-Sea, GBR

**Keywords:** critical care, methylene blue, amphetamine, cocaine, methemoglobinemia

## Abstract

Methaemoglobinaemia is a rare disorder characterized by increased levels of methaemoglobin, a form of haemoglobin with oxidized iron that cannot efficiently bind oxygen. This leads to inadequate oxygen delivery to tissues with various clinical manifestations from asymptomatic to severe persistent hypoxia, CNS symptoms, and cardiovascular collapse. Acquired methaemoglobinaemia is typically a sudden condition, often resulting from poisoning by specific drugs and compounds, which can potentially have fatal consequences. We present a case of a patient who came with severe methaemoglobinaemia due to intoxication with cocaine and amphetamine.

## Introduction

Over the past few years, there has been a surge in the prevalence of cocaine use in the United Kingdom, making it the second most frequently consumed drug. According to a survey conducted in 2018 and 2019, approximately 2.9% of adults between the ages of 16 and 59 reported using cocaine, amounting to around 976,000 individuals. Among young adults aged 16-24 years, cocaine ranked as the third most commonly used drug, with a usage rate of 6.2%, representing approximately 395,000 young adults. Notably, it trailed behind cannabis (17.3%) and nitrous oxide (8.7%) [1].

Methaemoglobinaemia occurs when haemoglobin oxidizes, forming methemoglobin (MetHb) and impeding oxygen transport, leading to tissue hypoxia in severe cases [2]. Methaemoglobinaemia is identified by elevated levels of MetHb in the bloodstream, a condition that arises when the iron component of haemoglobin undergoes oxidation, transitioning from the ferrous state (Fe2+) to the ferric state (Fe3+) and resulting in the formation of MetHb. This alteration renders MetHb unable to transport oxygen and causes a leftward shift in the oxygen dissociation curve. Consequently, this diminishes oxygen delivery, heightening the potential for tissue hypoxia [3].

Individuals in good health can withstand low levels of MetHb without encountering challenges. Cyanosis commonly serves as the initial indicator of an underlying methaemoglobinaemia. Hypoxemia, as assessed through pulse oximetry, is frequently noted as patients exhibit a misleading decline in peripheral oxygen saturation measurements. With an escalation in MetHb levels, symptoms such as dyspnea, headache, and dizziness may manifest. When MetHb surpasses 50%, there is a potential for developing arrhythmias, acidosis, seizures, coma, and, in extreme cases, fatality if levels exceed 70% [4].

Cocaine and its metabolites typically do not cause methemoglobinemia, but certain additives found in cocaine have been associated with this condition. Adulterants, intentionally added pharmacologically active substances in recreational drugs, are sometimes chosen to mimic drug effects for perceived quality and purity. However, the reasons for their inclusion are not always clear [5].

In situations where there is a temporal correlation between the onset of methaemoglobinaemia and cocaine use, it is crucial to investigate and exclude other simultaneous contributing factors [3]. Factors such as the use of e-cigarettes, consumption of water contaminated with nitrates/nitrites, intake of frozen items rich in nitrites/nitrates, certain foods like choy sum, herbal medications, topical and systemic dapsone used in anti-acne treatments, as well as the utilization of over-the-counter medications like eutectic mixture of local anaesthetics (EMLA) cream, have been identified as associations with methaemoglobinaemia [6-14].

We present a case of methaemoglobinaemia associated with mixed cocaine and amphetamine overdose.

## Case presentation

A 36-year-old male patient previously known to have asthma and depression presented to the Emergency Department (ED) with a depressed level of consciousness (Glasgow Coma Scale 3/15). History taken from the partner accompanying the patient suggested he was taking amphetamine and possibly snorting cocaine, with no other significant drug or social history. On arrival to the ED, the patient was hypoxemic (saturation of peripheral oxygen (SpO2) 80%) on high flow oxygen via a supraglottic airway, which was inserted by paramedics before arrival in ED, with a blood pressure of 105/60 and sinus tachycardia of 110/min. Arterial blood gas analysis showed metabolic acidosis with a pH of 7.30, partial pressure of carbon dioxide (pCO2) of 5.51 kPa, partial pressure of oxygen (PO2) of 40 kPa, lactate 6.4 mmol/L, bicarbonate (HCO3) 25.7 mmol/L, with 85.9% MetHb level. Laboratory investigations were sent and came back normal (Table 1). An X-ray was done and showed clear lungs with no aspiration pneumonitis. The patient was subsequently admitted to the intensive care unit, where he remained sedated on invasive mechanical ventilation, requiring low-dose vasopressor support.

**Table 1:** Blood investigations

Blood test	Value	Reference range
Haemoglobin	141 gm/L	130-180 gm/L
WBCs	12.7 x 10 ^9^/L	4.0-11.0 x 10 ^9^/L
Platelet	229 x 10 ^9^/L	150-400 x 10 ^9^/L
C-reactive protein	< 5 mg/L	<5 mg/L
Alanine transaminase	18 U/L	<50 U/L
Sodium	140 mmol/L	133-146 mmol/L
Potassium	3.9 mmol/L	3.5-5.3 mmol/L
Urea	3.1 mmol/L	2.5-7.8 mmol/L
Creatinine	82 umol/L	59-124 mmol/L

Based on the diagnosis of severe methaemoglobinaemia, treatment with intravenous methylene blue infusion was commenced at a dose of 2 mg/kg bolus. Another dose of 2 mg/kg of methylene blue was given after 60 minutes. On the second day of admission, MetHb levels dropped to 2.4%. As the ventilatory and cardiovascular support requirements were low, the decision was taken to extubate after appropriately responding when sedation was held. The patient was discharged from the intensive care unit on day two and from the hospital on day three of his admission.

## Discussion

Methemoglobinemia is diagnosed clinically by considering the patient's history and presenting symptoms such as hypoxemia that does not respond to supplemental oxygen and the observation of chocolate-coloured blood. Confirmation of the diagnosis involves arterial or venous blood gas analysis with co-oximetry, which identifies and quantifies the concentration and percentage of MetHb in the haemoglobin [15,16]. It is important to note that the severity of methemoglobinemia cannot be directly assessed using SpO_2_ measurements [17].

In the current case, the patient had a unique presentation of methaemoglobinaemia from a mixed overdose of cocaine and amphetamine. He presented with severe hypoxia that was managed with ventilatory support and methylene blue injection. 

Methylthioninium chloride, commonly known as methylene blue, is the preferred treatment for symptomatic methemoglobinemia patients. It functions as a substrate for nicotinamide adenine dinucleotide phosphate hydrogen (NADPH)-MetHb reductase, leading to the creation of reduced methylthioninium chloride. This reduced form serves as an electron donor, facilitating the reduction of Fe3+ back to Fe2+. The recommended dosage is 1-2 mg/kg, with the option for repetition if deemed necessary [13]. The reason to use methylene blue is that it activates NADPH MetHb reductase, which reduces methylene blue to methylene leucoblue, which transforms MetHb in Hb by a non-enzymatic mechanism [18].

Practitioners should be aware of the side effect profile of methylene blue. Benign side effects include green or blue discoloration of urine and patients should be forewarned. Our patient had a blue discoloration of the urine next day after the initiation of the treatment. Significant side effects are based on methylene blue, itself, being an oxidizing agent and an inhibitor of monoamine oxidase A (MAO-A). As an oxidizing agent, methylene blue can precipitate methaemoglobinaemia or haemolysis in high doses or when ineffectively reduced. Methylene blue administration in a patient taking serotonergic agents may predispose to serotonin syndrome [19].

Hyperbaric oxygen therapy, exchange transfusion, and ascorbic acid may be utilized if the treatment of methaemoglobin with methylene blue is ineffective or not recommended [18-19]. A high dose of vitamin C, which can be administered intravenously, is sometimes used to treat methaemoglobin, but it is generally ineffective [20].

## Conclusions

Methaemoglobinaemia is a rare condition characterized by elevated levels of methaemoglobin, a form of haemoglobin that contains oxidized iron, impeding the binding of oxygen and resulting in inadequate oxygen delivery to tissues. Acquired methaemoglobinaemia is often associated with drug poisoning, posing potential life-threatening risks. Symptoms vary depending on the percentage of methemoglobin, ranging from fatigue to severe manifestations such as altered consciousness and seizures. Unexplained symptoms like refractory hypoxia, a saturation gap, and blood with a chocolate hue may indicate methaemoglobinaemia.

This report presented a case of severe methaemoglobinaemia induced by a combination of cocaine and amphetamine intoxication. Maintaining vigilance regarding the possibility of methaemoglobinaemia is crucial for addressing it before it leads to devastating complications.
